# Microsatellite marker development for the rubber tree (*Hevea brasiliensis)*: characterization and cross-amplification in wild *Hevea* species

**DOI:** 10.1186/1756-0500-5-329

**Published:** 2012-06-25

**Authors:** Camila C Mantello, Fernando I Suzuki, Livia M Souza, Paulo S Gonçalves, Anete P Souza

**Affiliations:** 1 Centro de Biologia Molecular e Engenharia Genética (CBMEG) - Universidade Estadual de Campinas (UNICAMP), Cidade Universitária Zeferino Vaz, CP 6010, CEP 13083-970, Campinas, SP, Brazil; 2 Instituto Agronômico de Campinas (IAC), CP 28, Campinas, SP, CEP 13012-970, Brazil; 3 Departamento de Biologia Vegetal, Instituto de Biologia, Universidade Estadual de Campinas (UNICAMP), Cidade Universitária Zeferino Vaz, CP 6109, CEP 13083-970, Campinas, SP, Brazil

**Keywords:** *Hevea*, *H. brasiliensis*, Microsatellite, Characterization, Transferability

## Abstract

**Background:**

The rubber tree (*Hevea brasiliensis*) is native to the Amazon region and it is the major source of natural rubber in the world. Rubber tree breeding is time-consuming and expensive. However, molecular markers such as microsatellites can reduce the time required for these programs. This study reports new genomic microsatellite markers developed and characterized in *H. brasiliensis* and the evaluation of their transferability to other *Hevea* species.

**Findings:**

We constructed di- and trinucleotide-enriched libraries. From these two libraries, 153 primer pairs were designed and initially evaluated using 9 genotypes of *H. brasiliensis*. A total of 119 primer pairs had a good amplification product, 90 of which were polymorphic. We chose 46 of the polymorphic markers and characterized them in 36 genotypes of *H. brasiliensis*. The expected and observed heterozygosities ranged from 0.1387 to 0.8629 and 0.0909 to 0.9167, respectively. The polymorphism information content (PIC) values ranged from 0.097 to 0.8339, and the mean number of alleles was 6.4 (2–17). These 46 microsatellites were also tested in 6 other *Hevea* species. The percentage of transferability ranged from 82% to 87%. Locus duplication was found in *H. brasiliensis* and also in 5 of other species in which transferability was tested.

**Conclusions:**

This study reports new microsatellite markers for *H. brasiliensis* that can be used for genetic linkage mapping, quantitative trait loci identification and marker- assisted selection. The high percentage of transferability may be useful in the evaluations of genetic variability and to monitor introgression of genetic variability from different *Hevea* species into breeding programs.

## Findings

*Hevea brasiliensis* (Willd. ex Adr. de Juss.) Muell. -Arg., native to the Amazon rainforest, is a diploid (2n = 36, x = 9), perennial, monoecious and cross-pollinated tree species. It belongs to the genus *Hevea* and the botanical family Euphorbiaceae.

The genus *Hevea* comprises 11 inter-crossable species [1,2] (*H. benthamiana* Muell.- Arg., *H. brasiliensisH. carmagoana* Pires, *H. camporum* Ducke, *H. guianensis* Aubl, *H. microphylla* Ule, *H. nitida* Mart. ex-Muel.-Arg., *H. pauciflora* (Spruce ex-Benth.) Muell.-Arg., *H. rigidifolia* (Spruce ex-Benth.) Muell.-Arg., *H. spruceana* (Benth.) Muell.-Arg. and *H. paludosa* Ule), which have evolved in the Amazon rainforest over 100,000 years [3].

Of all the species in the genus *HeveaH. brasiliensis* is the most economically important, because it is the major source of natural rubber worldwide. Natural rubber is important mainly in the tire industry but also in many other sectors because it is flexible, resistant, impermeable to liquids and abrasion resistant [4]. These singular properties make natural rubber both complementary and competitive to synthetic rubber and furthermore superior to it in varied applications. As is the case for many other perennial trees, rubber tree breeding is time-consuming and expensive. An average duration of 20–25 years of field experiments in large areas is generally required to obtain a new cultivar with reasonably low risks [5].

Molecular markers, such as amplified fragment length polymorphisms (AFLPs), restriction fragment length polymorphisms (RFLPs), random amplified polymorphic DNA (RAPD), simple sequence repeats (SSRs or microsatellites) and single nucleotide polymorphisms (SNPs), have successively become increasingly important in plant breeding. These markers are efficient tools for the assessment of genetic diversity, the identification of quantitative trait loci (QTLs) and/or gene mapping, variety protection and marker-assisted selection (MAS) [6].

Of these markers, microsatellite markers are considered the most suitable for genetic studies, because they combine co-dominance and high polymorphism with abundance, locus specificity and uniform dispersion in plant genomes. Moreover, microsatellite markers can discriminate closely related individuals [7]. In addition, microsatellite analysis is inexpensive, high reproducible and highly transferable across related species.

Following biochemical markers such as isozymes [8], molecular markers have been developed and used since the middle of 1990’s for diversity studies [9,10], genetic mapping [11] and the identification of genetic loci implicated in the expression of agronomic traits in *H. brasiliensis*[11-14]. However, most of the markers used have been isozyme, RAPD, RFLP or AFLP markers.

To date, few studies using genomic microsatellites [10,15,16] or microsatellites from expressed sequence tags (EST-SSRs) have been published [6]. Accordingly, we present the development of genomic microsatellites using dinucleotide- and trinucleotide- enriched libraries, the characterization of these microsatellite markers in multiple accessions of *H. brasiliensis* and test their transferability in six other *Hevea* species.

## Results

### Microsatellite-enriched library analysis

Di- and trinucleotide-enriched libraries were constructed, and the clones from each library were sequenced (576 and 288 clones, respectively).

A total of 291 (50.5%) clones from the dinucleotide-enriched library contained microsatellite sequences. Since dinucleotide probes were used for the library enrichment, these motifs were the most abundant comprising 324 (90.2%) SSRs followed by 20 tetranucleotide (5.6%), 12 trinucleotide (3.3%) and 3 pentanucleotide (0.8%) (Table 1).

**Table 1:** Number of sequences and SSRs of each type in each library

	**Dinucleotide-enriched library**	**Trinucleotide-enriched library**
Number of sequences	576	288
Number of sequences with SSRs	291	62
Total number of SSRs	359	70
dinucleotide	324	13
trinucleotide	12	45
tetranucleotide	20	9
pentanucleotide	3	3

In the trinucleotide-enriched library, 62 (21.5%) clones contained microsatellites sequences. Trinucleotides were the most frequent motif with 45 (72.5%) SSRs followed by 13 dinucleotide (20.9%), 9 tetranucleotide (14.5%) and 3 pentanucleotide (4.8%) SSRs (Table 1).

For some sequences, both the dinucleotide- and trinucleotide-enriched libraries had more than one microsatellite, which explains the greater number of SSRs found compared to the number of sequences analyzed.

A total of 130 and 32 primer pairs were designed based on the di- and trinucleotide-enriched libraries, respectively. To remove possible redundancies with published SSRs, each sequence containing SSR was compared against GenBank database using BLASTN. Nine sequences, only from the dinucleotide library, were identical to previously published sequences, which already had primer pairs. These sequences were removed from this study.

Dinucleotide motifs have been reported as the most abundant type of microsatellite in plant genomes [17]. Recent studies relating to expressed sequence tags revealed that trinucleotide motifs are the most abundant motifs in ESTs in many plants, such as sugarcane [18], barley [19], grapes [20], rice [21], wheat [22] and citrus [23], whereas in other plants dinucleotide motifs are the most abundant in ESTs, such as kiwi [24], coffee [25] and apricot and peach [26] In rubber tree, Feng and co-workers analyzed all of the ESTs in NCBI database in search for microsatellites. They found that dinucleotide motifs were the most abundant and were three times more abundant than trinucleotides [6]. The low efficiency of the trinucleotide enrichment in our study could result from the low frequency of trinucleotide motifs in the rubber tree genome.

### Polymorphism analysis and cross-species transferability

In total, 153 specific primer pairs were designed. A total of 119 primer pairs produced good amplification products (Additional file 1: Table S1) and 90 of these products were polymorphic among a set of 9 rubber clones indicated in Table 2.

**Table 2:** **Genotypes of*H. brasiliensis*and six species of the genus*Hevea*used for characterization and transferability**

**Genotypes*H. brasiliensis***			**Other species of *Hevea***
RRIM 600	PB 311	IRCA 209	*H. guianensis*
RRIM 606	PB 346	IRCA 230	*H. rigidifolia*
RRIM 701^*^	PB 260^*^	IRCA 707	*H. nitida*
RRIM 729	PB 217^*^	IRCA 1159	*H. pauciflora* -(112 CNSG)
RRIM 728	PC 140	GT 1^*^	*H. pauciflora* -(116 CNSG)
RRIM 805	RRIC 100	PR255^*^	*H. benthamiana*
RRIM 809	IAC 306	RO 38^*^	*H. camargoana*
RRIM 913	IAC 307	Fx 4098	
RRIM 915	IAC 309	CMB 104^*^	
RRIM 937	IAC 313	CMB 114^*^	
RRII 105	IAC 318		
PB 233	IAC 500		
PB 235^*^	IRCA 27		

*: genotypes used to characterize all of the SSRs markers developed.

The observed and expected heterozygosities ranged from 0.1111 to 1 and 0.1111 to 0.9150, respectively, and the PIC values ranged between 0.0994 and 0.8496. The mean number of alleles was 4.46 (2–9 alleles).

Of the 90 polymorphic markers we chose 46 SSRs for characterization among a set of 36 genotypes of *H. brasiliensis* (Table 2). The PIC values of these markers ranged from 0.097 to 0.8339, and the observed and expected heterozygosities ranged from 0.0909 to 0.9167 and 0.1387 to 0.8629, respectively. The mean number of alleles was 6.4 (2–17 alleles) (Table 3).

**Table 3:** Characterization of the 46 polymorphic SSR markers

***Primer***	**NA**	**size range (bp)**	**H**_**e**_	**H**_**o**_	**PIC**	**NA**
	**A**					**B**
HB 31	6	182-173	0.753	0.6875	0.7001	12
HB 32	6	256-238	0.6057	0.3243	0.524	12
HB 33	9	188-171	0.6808	0.4	0.6388	10
HB 35	3	159-156	0.3882	0.4054	0.3226	5
HB 36	7	239-217	0.6279	0.6111	0.557	16
HB 37	3	161-154	0.6133	0.3235	0.5325	3
HB 41	6	141-173	0.4855	0.3611	0.459	8
HB 42	7	203-216	0.5313	0.5833	0.4954	10
HB 43	10	219-265	0.7531	0.5405	0.7133	15
HB 45	17	155-238	0.856	0.8333	0.8273	21
HB 47	8	152-201	0.6311	0.4722	0.566	14
HB 50	3	196-210	0.2289	0.1944	0.2124	8
HB 51	2	196-201	0.4909	0.1515	0.3666	7
HB 53	8	204-223	0.7871	0.7568	0.7439	13
HB 54	10	168-203	0.6708	0.5294	0.6201	10
HB 55	9	168-192	0.8	0.4571	0.7645	10
HB 57	9	147-176	0.7169	0.6471	0.6831	12
HB 60	5	148-161	0.3154	0.3514	0.2985	6
HB 61	6	150-172	0.7175	0.9167	0.6662	11
HB 62	5	122-112	0.415	0.1081	0.3888	5
HB 63	8	198-236	0.7553	0.5135	0.7082	15
HB 64	8	146-199	0.6221	0.4444	0.5914	12
HB 66	3	273-279	0.2273	0.25	0.2085	4
HB 68	10	138-167	0.8058	0.8286	0.7636	14
HB 69	10	139-172	0.6962	0.6471	0.6426	10
HB 70	5	155-167	0.6928	0.8	0.6281	6
HB 71	2	186-188	0.1037	0.1081	0.097	2
HB 73	6	206-228	0.7754	0.8056	0.729	8
HB 77	4	135-153	0.1788	0.0811	0.1701	8
HB 78	8	186-215	0.7996	0.8286	0.7605	10
HB 81	9	187-220	0.7469	0.5333	0.6979	13
HB 82	4	175-168	0.5572	0.6757	0.4489	8
HB 83	4	181-165	0.5271	0.7143	0.4631	4
HB 92	7	255-229	0.773	0.7576	0.7246	7
HB 95	6	254-225	0.5742	0.5946	0.4793	12
HB 98	3	190-180	0.2787	0.3143	0.2535	4
HB 100	10	250-214	0.803	0.8649	0.7657	16
HB 101	2	142-132	0.2166	0.2432	0.1908	4
HB 102	4	198-157	0.2714	0.2973	0.2568	10
HB 103	2	164-161	0.1037	0.1081	0.097	2
HB 104	5	173-154	0.6711	0.375	0.6119	14
HB 105	13	258-176	0.8629	0.9091	0.8339	21
HB 106	5	210-230	0.5855	0.0909	0.4975	6
HB 109	6	186-210	0.1387	0.1143	0.135	7
HB 110	9	273-256	0.8322	0.6667	0.7981	11
HB 117	8	152-190	0.8052	0.7222	0.7643	12

NA/A, number of alleles in the 36 *H. brasiliensis* clones; NA/B, the total number of alleles in the 36 *H. brasiliensis* clones and in the other *Hevea* species; bp, product size range in base pairs; He, expected heterozygosity; Ho, observed heterozygosity; PIC, polymorphism information content.

Six other species from the genus *Hevea* (*H. guianensis*, *H. rigidifolia*, *H. nitida*, *H. pauciflora*, *H. benthamiana and H. camargoana*) being two different genotypes of *H. pauciflora*, were used to evaluate the transferability of the markers (Table 2). All loci were tested under the same PCR conditions used for *H. brasiliensis*.

Of the 46 loci tested, 40 (87%) were amplified for *H. guianensis* and *H. pauciflora*- (112CNSG), 39 (85%) were amplified for *H. camargoana*, *H. nitida* and *H. pauciflora*-(116CNSG) and 38 (82%) were amplified for *H. benthamiana* (Table 4).

**Table 4:** **Cross-amplification of the 46 polymorphic SSRs markers among the other*Hevea*species**

***Primer***	***H. guianensis***	***H. rigidifolia***	***H. benthamiana***	***H. camargoana***	***H. nitida***	***H. pauciflora*-*(112 CNSG)***	***H. pauciflora*-*(116CNSG)***
HB-31	+	+	+	+	+	-	+
HB-32	+	+	+	+	+	+	+
HB-33	+	-	+	+	+	+	+
HB-35	+	+	+	+	+	+	+
HB-36	+	+	+	+	+	+	+
HB-37	+	-	-	+	-	-	-
HB-41	+	+	+	+	+	+	+
HB-42	+	+	+	-	-	+	-
HB-43	+	+	+	+	+	+	+
HB-45	+	+	+	+	+	+	+
HB-47	-	+	+	+	+	+	+
HB-50	-	+	+	+	+	+	+
HB-51	+	+	+	+	+	+	-
HB-53	+	+	+	+	+	+	+
HB-54	-	-	-	-	-	-	-
HB-55	+	+	-	-	-	+	+
HB-57	+	+	+	+	+	+	+
HB-60	+	+	+	+	+	+	-
HB-61	+	+	+	+	+	+	+
HB-62	-	-	-	-	-	-	-
HB-63	+	+	+	+	+	+	+
HB-64	+	+	+	+	+	+	+
HB-66	+	+	+	+	+	+	+
HB-68	-	+	+	+	+	+	+
HB-69	-	-	-	-	-	-	-
HB-70	+	+	+	+	+	+	+
HB-71	+	+	+	+	+	+	+
HB-73	+	+	+	+	+	-	-
HB-77	+	+	+	+	+	+	+
HB-78	+	+	+	+	+	+	+
HB-81	+	-	+	+	+	+	+
HB-82	+	+	+	+	+	+	+
HB-83	+	+	+	+	+	+	+
HB-92	+	-	+	+	+	+	+
HB-95	+	+	+	+	+	+	+
HB-98	+	+	+	+	+	+	+
HB-100	+	+	+	+	+	+	+
HB-101	+	+	+	+	+	+	+
HB-102	+	+	+	+	+	+	+
HB-103	+	+	-	-	+	+	+
HB-104	+	+	+	+	+	+	+
HB-105	+	+	+	+	+	+	+
HB-106	+	-	+	-	-	+	+
HB-109	+	+	+	+	+	+	+
HB-110	+	+	+	+	+	+	+
HB-117	+	+	+	+	+	+	+

For most of the SSR loci, which cross-species amplification were tested, the number of alleles found in the *H. brasiliensis* genotypes in conjunction with the tested species (from 2 to 21, with a mean number of 9.5) was larger when the same loci were analyzed in *H. brasiliensis* alone (Table 3) which means that other species revealed some novel alleles.

Three primers pairs, HB54, HB62 and HB69, did not produce amplification products for the six other species tested, whereas 27 loci were amplified for all species (Table 4).

Saha and co-workers first observed SSR cross-amplification in *H. benthamiana* and *H. spruceana*[27]. Together with the cross-fertility potential, this high SSR transferability supports the consideration of the *Hevea* genus as a species complex with moderate differentiation among the species. These aspects appear to be favorable for genetic introgressions using other *Hevea* species to the rubber breeding population, which is mainly based on *H. brasiliensis*.

### Locus duplication

Members of the Euphorbiaceae family have a basic number of chromosomes between 6 and 11; thus, any species with more than this number of chromosomes could be amphidiploid in origin [28,29]. Similar to cassava, plants in the genus *Hevea* have 36 chromosomes and behave as diploid. In these species, it has been assumed that n = 18 and the basic number of chromosomes is x = 9 [30,31].

In different *Hevea* species, chromosomes mainly formed bivalents and tetravalents are rarely produced as a result of pairing between non-homologous chromosomes during prophase I and metaphase I of meiosis [31,32].

Moreover, cytogenetic studies revealed two distinct loci on two different chromosomes, bearing the same18S-5.8 S-25 S rDNA sequence which may have arisen by the hybridization of two unknown diploid species (n = 9), thus suggesting a possible allotetraploid origin, however no potencial diploid ancestor has been described to date [29].

Locus duplication, as revealed by molecular markers, had been reported in *H. brasiliensis* 11] and *H. guianensisH. rigidifolia* and *H. pauciflora*[15].

In *H. brasiliensis*, we observed locus duplication for the markers HB45 and HB109 for clones RRIM 701 and Fx4098, respectively. Cases of locus duplication were observed in all *Hevea* species tested, with the exception of *H. rigidifolia*. We also observed locus duplication of the HB36, HB68, HB100 and HB105 markers in *H. nitida*; HB68 and HB105 markers in *H. benthamiana*; HB101 and HB105 in *H. guianensis*. The marker HB105 exhibited one duplicated locus for *H. pauciflora* (112 CNSG), *H. pauciflora* (116 CNSG) and *H. camargoana*.

**Figure 1:**
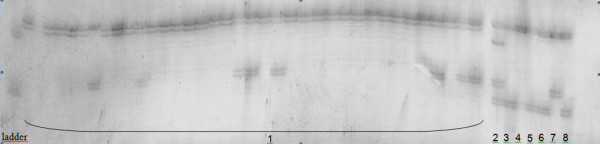
**A 6% denaturing silver-stained polyacrilamyde gel for the characterization of the HB101 marker.** PCR product for the HB101 marker in the 36 genotypes of *H. brasiliensis* and the 6 other species of *Hevea* which more than 2 alleles can be observed for *H. guianensis*. (**1**) *H. brasiliensis*; (**2**) *H. guianensis*; (**3**) *H. rigidifolia*; (**4**) *H. nitida*; (**5***) H. pauciflora* - (112 CNSG); (6) *H. pauciflora* - (116 CNSG); (**7**) *H. benthamiana*; and (**8**) *H. camargoana.*

Although we have not been mapped the loci we cited as duplicated in the wild species, we observed more than 2 alleles (excluding the stutter bands) in the same base pair range compared with the other genotypes used for characterization (Figure 1). The presence of allele duplication, as determined by polyacrylamide gel electrophoresis suggests that the loci are duplicated for the species involved.

Although locus duplication had been described in *H. brasiliensis*, *H. guianinsis*, *H. rigidifolia* and *H. pauciflora*, this is the first report for *H. benthamiana*, *H. camargoana* and *H. nitida*.

## Conclusion

Herein, we report the development of new SSR markers for *H. brasiliensis*, representing a powerful resource for genetic diversity studies and genetic breeding techniques, such as molecular genetic mapping, QTLs identification and MAS.

Due to observed high percentage of cross-amplification and the absence of reproductive barriers between the species within this genus, these markers may be important tools to monitor the genetic variability from other *Hevea* species into the current breeding programs.In addition, these SSR markers can be helpful for the identification of important agronomic characteristics in different *Hevea* species.

## Methods

### Plant material and DNA extraction

We used 36 genotypes of *H. brasiliensis* to characterize the SSRs (Table 2). These genotypes were kindly provided by the Agronomic Institute of Campinas (IAC) and Michelin Plantation (Brasil). We also used 6 other species of the genus *Hevea* (*H. guianensisH. rigidifoliaH. nitidaH. paucifloraH. benthamiana* and *H. camargoana*) that were kindly provided by the Brazilian Agricultural Research Corporation (EMBRAPA - Amazônia Ocidental) in Manaus, AM to test the transferability of the SSRs (Table 2). The genomic DNA samples were extracted from lyophilized leaf tissues using a modified CTAB method [33] and their quality and quantity were assessed using 1% agarose gel electrophoresis.

### Construction of microsatellite-enriched libraries and sequence analysis

The microsatellite-enriched libraries for *H. brasiliensis* were constructed using the RRIM 600 clone according to the methodology described by Billote and co-workers [34]. The DNA samples were digested with AFAI and enriched using streptavidin magnetic-coated beads (Streptavidin MagneSphere Paramagnetic Particles, Promega, Madison, WI) and (CT)_8_ and (GT)_8_ biotinylated microsatellite probes for the dinucleotide-enriched library or (ATC)_8_ and (CCT)_8_ for the trinucleotide-enriched library.

Selected DNA fragments were amplified by PCR and then cloned into the pGEM-T vector (Promega, Madison, WI). Competent XL1-blue *Escherichia coli* cells were transformed with the recombinant plasmids and cultivated on agar medium containing ampicillin and 100 μg/ml of X-galactosidase. The clones containing the insert were sequenced using the Big Dye Terminator v3.1 Cycle Sequencing Kit (Applied Biosystems, Foster City, CA) and an automated ABI 377 sequencer (Applied Biosystems, Foster City, CA).

All the sequences obtained were aligned, edited and eliminated if redundant using SeqMan (DNASTAR, Madison, WI). The sequences were also evaluated using Microsat software (A. M. Risterucci, CIRAD, personal communication), which removes the adapters and verifies the presence of restriction sites.

The microsatellites were identified using a specific research tool, SSRIT – (Simple Sequence Repeat Identification Tool) [35]. As a criterion for the SSR selection, the sequences that showed at least five dinucleotide repeats; four trinucleotide repeats; and three tetra-, penta- and hexanucleotide repeats were selected. Primers complementary to the sequences flanking the microsatellites were designed using Primer Select Program (DNAStar, Madison, WI) and Primer 3 [36]. To eliminate possible redundancies with the published SSRs, each sequence containing microsatellite was compared against the GenBank database using BLASTN. The redundant sequences which already had primer pairs published were eliminated.

### PCR amplification

The microsatellite fragments were PCR amplified in a 15 μl reaction containing 25 ng template DNA, 0.5 μM each primer, 100 μM each dNTP, 1.5 mM MgCl_2_, 20 mM Tris–HCl, 50 mM KCl and 0.5 U Taq DNA Polymerase. The PCR amplifications were performed with the following four programs: **(1)** initial denaturation at 94°C for 4 min, 30 amplification cycles (1 min at 94°C, 45 s at the specific annealing temperature and 1 min at 72°C), and a final extension at 72°C for 10 min; **(2) TD-1**, initial denaturation for 3 min at 94°C, 10 amplification cycles with a 0.5°C decrease in annealing temperature per cycle starting at 55°C or 60°C (94°C for 1 min, 60°C or 55°C for 30 s, and 72°C for 1 min 15 s); followed by 20 cycles with annealing at 50°C (94°C for 1 min, 50°C for 30 s and 72°C for 30 s) and a final elongation step at 72°C for 10 min; **(3) TD-2**, previously described by Le Guen and co-workers [10]; and **(4) TD-3**, initial denaturation at 94°C for 2 min, 2× 10 cycles with a 1°C decrease in annealing temperature per cycle from 65°C to 55°C (94°C for 1 min, 65°C for 1 min and 72°C for 1 min), followed by 18 cycles at 55°C (94°C for 1 min, 55°C for 1 min and 72°C for 2 min), and a final elongation step at 72°C for 5 min.

The amplification products were resolved by electrophoresis through 3% agarose gels prior to vertical electrophoresis using 6% denaturing polyacrylamide gels and were subsequently silver stained [37]. The product sizes were determined by comparison with a 10 bp DNA ladder (Invitrogen, Carlsbad, CA).

### Analysis

The allelic polymorphic information content of each SSR was calculated using the formula$PIC=1-\sum_{i=1}^{n}p_{i}^{2}-\sum_{i=1}^{n}\sum_{j=i+1}^{n}2p_{i}^{2}p_{j}^{2}$, where n is the number of alleles of the marker among the set of genotypes used for characterizing the SSR polymorphism, and pi and pj are the frequencies of alleles i and j. The observed and expected heterozygosities were calculated using the TFPGA program [38].

## Competing interests

The authors declare that they have no competing interests.

## Authors’ contributions

CCM and FIS developed the microsatellite-enriched libraries, performed the computational microsatellite identification, designed the flanking primers and performed the microsatellite marker validation. CCM performed the statistical analysis and drafted the manuscript. LMS participated in the design and implementation of the study and the microsatellite identification. APS and PSG conceived the study and participated in its design and coordination. APS helped to draft the manuscript. All of the authors read and approved the final manuscript.

## Supplementary Material

Additional file 1 ** Table S1.** Characterization of the developed SSR markers. The table presents the 119 SSR markers developed for *H. brasiliensis*, including the primers sequence, annealing temperature, number of alleles, expected size in base pair, allelic range, observed and expected heterozigosity and polymorphism information content. The nine accession indicated in Table 1 were used for the SSR characterization. Click here for file
